# Effect of multimorbidity on quality of life in adult with cardiovascular disease: a cross-sectional study

**DOI:** 10.1186/s12955-017-0820-8

**Published:** 2017-12-08

**Authors:** Bijan Shad, Asieh Ashouri, Tolou Hasandokht, Fatemeh Rajati, Arsalan Salari, Moona Naghshbandi, Fardin Mirbolouk

**Affiliations:** 1 Department of Cardiology, Cardiovascular Diseases Research Center, Heshmat Hospital, School of Medicine, Guilan University of Medical Sciences, Rasht, Iran; 2 School of Health, Guilan University of Medical Sciences, Rasht, Iran; 3 Department of Community Medicine, School of Medicine, Guilan University of Medical Sciences, Rasht, Iran; 4 Departments of health education and promotion, School of Public Health, Kermanshah University of Medical Sciences, Kermanshah, Iran

**Keywords:** Multimorbidity, Quality of life, Comorbidity, Coronary artery disease

## Abstract

**Background:**

The aim of present study was to describe the effect of multimorbidity on Health-Related Quality of Life (HRQoL) in patients with coronary artery disease (CAD).

**Methods:**

A cross-sectional study with a simple sampling method of 296 patients undergoing coronary artery bypass surgery in a referral hospital of the northern part of Iran was conducted between April, 2015 and September, 2016. Multimorbidity was defined as the presence of at least two chronic diseases based on self-reporting and medical records. HRQoL was measured using the 36-item short form (SF-36) health status survey. We used analysis of variance (ANOVA) to assess the effect of multimorbidity on mental and physical component of HRQoL.

**Results:**

Approximately, 69% of CAD patients had at least one other disease like diabetes or hypertension. Patients without multimorbidity compared with patients with multimorbidity were significantly older (*p* = 0.012) and more educated (*p* = 0.002). Both physical and mental component score of HRQoL was better in patients without any morbidity (48.82 vs. 43.93 with 95%CI of mean difference: 3.37–6.42 and 54.85 vs. 50.44 with 95% CI of mean difference: 1.68–7.15, respectively). Both physical and mental component score was significantly lower in female and lower educated patients (physical mean score 43.07 vs. 46.54 with *P* = .001 and 42.53 vs. 46.82 with *P* < .001 and mental mean score 49.98 vs. 52.65 with *P* = .055 and 49.80 vs. 52.75 with *P* = .022 for sex and education, respectively). Also, two-way ANOVA showed that regards to morbidity, physical component score was grater in patients with lower education level than higher education level (*P* < .001).

**Conclusion:**

The findings of this study suggest that women, lower education level and overweight reported lower quality of life. HRQoL is affected by multimorbidity among CAD patients specially in less educated.

## Background

Coronary artery bypass graft (CABG) surgery is known as an effective treatment for advanced coronary artery disease (CAD) with high survival rate in the early years of surgery [1]. Evidence showed that CABG surgery can reduce symptoms and disability [2]. Patients with CAD needs continuous care and requires multiple risk factor like diabetes (DM), hypertension (HTN), and hyperlipidemia management strategies. Coexistence of two or more chronic conditions in one patient was defined as multimorbidity (MM) [3], which have negative effect on physical and mental function and higher need to major treatment [4]. According to world health organization definition of health, quality of life must be considered as an substantial health outcome in every disease management. Health-Related Quality of Life (HRQoL) measurement provides an acceptable and valid method for assessing the impact of disease on patients’ function, activity and well-being [5]. Previous studies showed a lower quality of life among subjects with CAD [6, 7] and also when compared with the general population [8]. CABG is a known treatment for CAD could improve health-related quality of life (HRQoL), and increase survival [8, 9]. However, recent studies discuss about some factors could change the quality of life after CABG [10, 11]. Luc Noyez in a review article mentioned about considering demographic factors and comorbidity in evaluation of HRQoL after cardiac surgery [10]. Several factors such smoking,, high alcohol intake, low socioeconomic state, presence of other disease, low education level and overweight were found to affect improvement of quality of life after CABG [12, 13]. Moreover, Impaired HRQoL in patients with MM has been described, previously [14, 15]. Studies about the impact of multimorbidity on HRQoL after cardiac surgery is limited [16]. Knowing the HRQoL in CAD patients with MM could help clinicians to design and development of appropriate health programs.

The aim of this study was to describe the effect of multimorbidity on HRQoL in patients 5 years post CABG.

## Methods

### Study design

This is a cross sectional study evaluating the health status of CAD patients after 5 years coronary artery bypass graft. Health survey conducted between 2015 and 2016 in a teaching hospital located in the northern part of Iran.

### Data collection

A simple sampling of patients older than 20 years underwent CABG in 2010 who were alive on discharge time were drawn from hospital records (*n* = 354). Patients were invited to participate in the study through telephone call. A specific visit time was scheduled for all participants. Those patients who refused to participation after two times invitation were excluded from the study (*n* = 7).

**Fig. 1:**
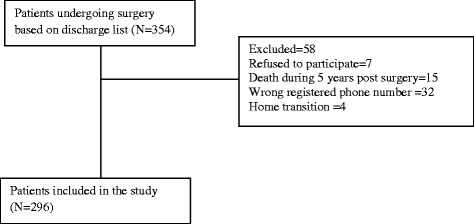
A chart reporting the flow of participants through each stage of the study

Other reasons for attrition were including; death during 5 years (15), wrong registered phone number (32), home transition (4) (Fig. 1).

Subjects were requested to bring all medications currently taken, together with their medical reports.

On the day of the study visit, after obtaining informed consent, structured interview were performed by a trained research assistant. Questions were about demographic characteristics including age, gender, Living location (rural or city), education level (less than 12 years of education or higher) and smoking status. Data on any recent cardiac symptoms like chest pain and exertional dyspnea post surgery were assessed by a cardiologist. Cardiac symptoms were presented as yes or no. Anthropometric parameters like height and weight were measured in light clothes without shoes by a precalibrated digital SECA scale and portable stadiometer by a research assistant. We calculated body mass index (BMI) for study population as weight in kg divided by squared height in m. Normal weight was defined as BMI < 25, overweight as BMI 25–29.9, and obesity as BMI ≥ 30 kg/m^2^. Medical history including diabetes and hypertension was considered present according to patients self report and medications. . We categorized multimorbidity to 1) CAD without any morbidity (*n* = 91), 2) CAD plus diabetes (*n* = 30), 3) CAD plus hypertension (*n* = 76), 4) CAD plus diabetes and hypertension (*n* = 99).

All subjects were interviewed to assess HRQoL by 36-item short form (SF-36) health status survey. The SF-36 questionnaire measured daily functioning abstracted into two summary measures: physical component summary (PCS) and mental component summary (MCS) scales [17]. PCS scale included five domains including physical functioning, role physical, bodily pain, general health, and vitality. MCS scale consist of general health, vitality, social functioning, role emotional, and mental health. Total score range for each item was varied from 0 to 100 scales from worst to best. Persian version of questionnaire was translated and validated by Montazeri et al. [18]. A minimum difference in three to five points is considered clinically important based on a previous review [19].

### Statistical analysis

Mean (SD, range) and frequency (percentage) was reported to describe the study population. Normal distribution assumption was checked by skewness and Kurtosis criteria (lower than 1) and Kolmogrov-Smirnov test. Independent sample t-test and Chi-square test was performed to compare continuous and categorical variables, respectively. Pearson correlation was used to estimate the correlation between variables. Mean score of physical and mental component were obtained for patients with and without morbidity and groups according to multimorbidity (without morbidity, with diabetes only, with hypertension only and with comorbidity of diabetes and hypertension). One-way analysis of variance (ANOVA) was performed to compare physical or mental component score between groups and Tukey HSD test was used for post hoc pair comparisons. In the non-homogeneity of variances condition, Welch F ratio statistics and Games-Howell post hoc test was used to test the difference of physical or mental component score between comorbidity groups. Two-way ANOVA were performed to compare component of quality of life score between co-morbidity groups adjusted for demographic variables. Data were analyzed by SPSS software version 18 and *P* < 0.05 was considered statistically significant.

## Results

Patients’ characteristics are shown in Table 1. In total, 296 patients aged between 44 and 89 years (mean age 62.5 ± 8.1) were included in the study. Two hundred and one (68%) were male. Ninety one patients (31%) had no comorbid chronic conditions. Compared with CAD patients without multimorbidity, those with multimorbidity were younger (mean age 64.03vs. 62.90 years), more female and more with lower education level.

**Table 1:** Patients characteristics in total and by co-morbidities with CAD groups

		Without co-morbidity	With co-morbidity					
	Total (*n* = 296)	(n = 91, 30.7%)	Total (*n* = 205, 69.2%)	DM only (*n* = 30, 10.1%)	HTN only (*n* = 76, 25.6%)	DM + HTN (*n* = 99, 33.4%)	P*	P**
Sex, no. (%)							<.001	<.001
Male	201(68)	78(39)	123(61)	25(12)	52(26)	46(23)		
Female	95(32)	13(14)	82(86)	5(5)	24(25)	53(56)		
Living location, no. (%)							.419	.224
Urban	238(80)	77(32)	161(68)	23(10)	57(24)	81(34)		
Rural	58(20)	14(24)	44(76)	7(12)	19(33)	18(31)		
Education, no. (%)							.001	.002
< diploma	96(32)	18(19)	78(81)	5(5)	30(31)	43(45)		
> = diploma	200(68)	73(37)	127(63)	25(13)	46(23)	56(28)		
Symptom, no. (%)							.943	.559
Yes	97(33)	32(33)	65(67)	10(10)	24(25)	31(32)		
No	199(67)	59(30)	140(70)	20(10)	52(26)	68(34)		
Smoking, no. (%)							<.001	<.001
Yes	83(28)	36(43.3)	47(56.6)	8(9.6)	23(28)	16(19)		
No	213(72)	55(28.5)	158(74)	22(10)	53(25)	83(39)		
BMI***, mean (SD)	28.37(4.70)	26.17(3.58)	29.32(4.82)	28.55(2.45)	28.41(4.88)	30.23(5.12)	<.001	<.001
Normal	76(26)	36(47)	40(53)	2(3)	18(24)	20(26)		
Overweight	118(41)	40(34)	78(66)	22(19)	26(22)	30(25)		
Obese	95(33)	11(12)	84(88)	3(3)	32(34)	49(51)		
Age, mean (SD)	62.52(8.10)	64.03(9.45)	63.33(8.36)	62.85(5.48)	60.03(7.21)	62.90(8.14)	.065	.012

*P-value was reported for comparison between four co-morbidity groups (without co-morbidity, DM, HTN, DM+HTN)

**P-value was reported for comparison between patients with and without co-morbidity

***BMI data was missing for 7 (2%) of patients

Descriptive statistics of quality of life scores are presented in Table 2. Both physical and mental component score of quality of life was significantly lower in patients with multimorbidity compared with those without (48.82 vs. 43.93 with 95%CI of mean difference: 3.37–6.42 and 54.85 vs. 50.44 with 95%CI of mean difference: 1.68–7.15 for physical and mental component score, respectively) (table 2). There was a weak relation between physical and mental component score (*r* = .206, *P* < .001).

**Table 2:** Physical and mental component score in total and by comorbidities with CAD groups

	Total (*n* = 296)	None (*n* = 91)	DM only (*n* = 30)	HTN only (*n* = 76)	DM + HTN (*n* = 99)	*P*
Physical component						<.001*
Mean (SD)	45.43(7.84)	48.82(4.81)	41.37(10.56)	45.65(7.39)	43.37(8.30)	
Min-max	23.54–60.00	36.10–57.17	23.54–54.11	25.88–56.31	26.83–60.00	
Mental component						.018**
Mean (SD)	51.80(11.21)	54.85(10.82)	50.23(12.17)	49.99(11.48)	50.84(10.65)	
Min-max	29.29–74.53	29.29–74.53	31.59–68.32	33.34–73.34	31.50–70.65	

*P*-value was reported for comparison between patients with and without comorbidity

*There was a significant difference between none co-morbidity group and DM (*P* = .004), HTN (*P* = .009) and DM + HTN (*P* < .001) based on Games-Howell post hoc test

**There was a significant difference between none co-morbidity group and HTN (*P* = .026) based on Tukey HSD post hoc test

Physical component score comparison between different multimorbidity groups showed that there was a significant difference between non multimorbidity group and each of DM (*P* = .004), HTN (*P* = .009) and DM + HTN (*P* < .001) groups. But there was no any differences between DM, HTN and DM + HTN pairs groups (*P* > .20 for all other comparisons, based on Games-Howell post hoc test). Also, pairwise comparison of mental component score between groups showed that there was only a significant difference between CAD group without morbidity and those with HTN only group (*P* = .026). There were no other significant differences between DM, HTN and DM + HTN pairs groups (*P* > .05 for all other comparison based on Tukey post hoc test).

Assessing relationships between demographic characteristics and physical and mental component score didn’t showed any significant association; for age (*r* = −.07 with *P* = .241 and *r* = −.03 with *P* = .636, respectively), living location (*P* = .796 and *P* = .123, respectively), smoking status (*P* = .716 and *P* = .880, respectively) and cardiac symptom (*P* = .452 and *P* = .535, respectively). But, Physical component score was related to the patients sex (*P* = .001), educational level (*P* < .001) and BMI (*P* = .044).

Women reported statistically significant lower PCS than men (mean score 43.07 vs. 46.54, 95% CI of mean difference: 1.40–5.54, P = .001). Also, physical component score in patients with higher educational level was grater than those with low education level (mean score 46.82 vs 42.53, 95% CI of mean difference: 2.17–6.41, *P* < .001). Overweight and obese patients reported lower PCS score when compared to normal weight. The mean score of PCS in normal, overweight and obesity were 48.23, 45.91, and 42.86 respectively, P = .044).

Post hoc test showed patients with obesity had lower physical component score compared with normal or overweight patients (95% CI of mean difference: 2.94–7.81 with *P* < .001 and 95%CI of mean difference: .48–5.63 with *P* = .015, respectively). There was no significant difference between normal and overweight patients regard to physical component score (95%CI of mean difference: −.05–4.69 with *P* = .057).

Mental component score was related to the patients sex (*P* = .052) and educational level (*P* = .022) but not related to the BMI (*P* = .918).Mental component score was significantly lower in female (mean score 49.98 vs. 52.65, 95%CI of mean difference: −.06–5.41, P = .052), and in patients with lower education level (mean score 49.80 vs. 52.75, 95%CI of mean difference: .44–5.47, P = .022).

**Fig. 2:**
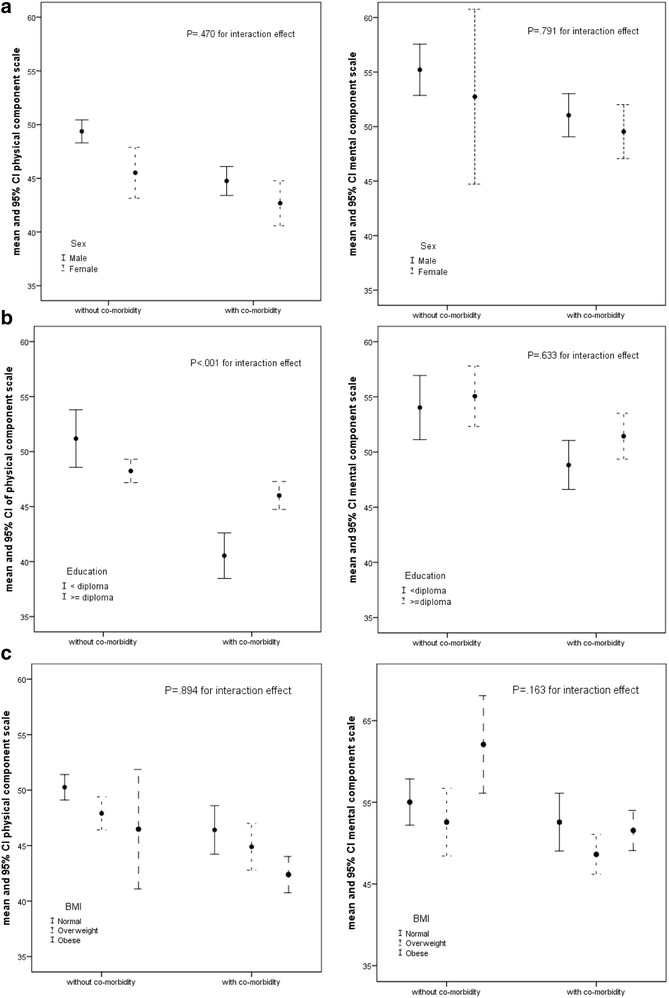
Mean and 95% confidence interval of physical and mental component score based on patient‘s comorbidity group and sex (**a**) or educational level (**b**) or BMI categories (**c**)

Regards to both physical and mental component score, there were no interaction between patients with and without multimorbidity groups and sex (*P* = .470 and *P* = .791, respectively) or BMI level (*P* = .894 and *P* = .163, respectively). It means that there was no significant difference in the HRQoL score among patients with multimorbidity between sex or BMI category (Fig. 2).

For mental component score, there was no interaction between with and without multimorbidity groups and education level (*P* = .633). However for physical component score, two-way ANOVA showed an interaction between patients with and without multimorbidity group and educational level (*P* < 0.001, Fig. 1). So, in patients with lower education level, decrease in the physical component score was grater and clinically significant (mean score 40.54 vs. 51.18, 95% CI of mean difference: 7.39–13.91, *p* < 0.05), but in patients with higher education level, decreasing the physical component score was low and not clinical significant (mean score 46.01 vs. 48.24, 95%CI of mean difference: .59–3.87, *p* < 0.05) (Fig. 1).

## Discussion

The result of our study showed that 69% of study population had at least one morbidity condition along with CAD. The most common morbidity was hypertension among CAD patients. Hypertension is a one of the important disease in patients with CAD [20].In the present study, patients with multimorbidity were slightly older than those without multimorbidity. Patients with several risk factors were more at risk of coronary artery disease [21].According to a recent systematic review, prevalence of multimorbidity increase in older people [22]. And also parallel with previous studies, multimorbidity was more common among women [3, 23, 24] and less educated people [25]. The prevalence of multimorbidity in our hospital base data was higher than general population in Ramezankhani study [26]. In the recent study, about 35–40% of general population had multiple cardiovascular risk factors like hypertension or diabetes [26]. Although the high prevalence of multiple disease in patients admitted in hospitals is expected. But Agborsangaya and colleague [24] showed co occurrence of multiple disease is prevalent in 18 years and over in the general population. Hence, development and implantation of prevention program for general population seems to be important.

Physical and mental component of quality of life in CAD patients with multimorbidity were worse when compared to those without. Furthermore, in the present study CAD patients without any morbidity reported better PCS compared to CAD plus HTN, or plus DM or both. Also, we observed a reduction in the score of mental component of HRQoL among CAD group with hypertension when compared to CAD without any morbidities. Similarly, the literature showed cardiomethabolic disorder was associated with reduced score in physical component of HRQoL rather than mental component [27]. Interestingly, we didn’t detect any dose response relationship between increasing in the number of morbidity and PCS as well as MCS. Although, a recent systematic review reported an association between number of multimorbidity and clinical outcome in patients with cardiovascular disease [16]. But, to the best of knowledge there is not any study focusing on such association with quality of life in cardiovascular disease. However, several studies showed the negative effect of increasing in the number of morbidity and HRQoL in diabetic patient [28, 29].

In our study, lower physical and mental component of HRQoL were observed in women and less educated CAD patients, consistent with previous studies [5, 30]. We found in lower educated group, presence of multimorbidity have clinically important effect on PCS. While in higher educated patients, we didn’t find any clinically effect of multimorbidity on PSC. which is confirmed by Yulian Zhanget al study [31]. This issue indicated the importance of patient’s knowledge about lifestyle behavior and self management [32]. Similarly, Choowattanapakorn et al. in a multicenter study showed self-care behavior was a predictive factor of PCS in 345 diabetic patients. When patients more know about causes of disease and treatment, they can better modify their health behaviors. According to the previous evidence, women more express such symptoms related to energy and fatigue than do men [33]. On the other hands, Sylvie S. L et al. in a multicenter, prospective cohort study showed better social function score in men compared to women [34]. Moreover, in the present study patients with higher BMI level reported lower physical component of quality of life. But, we didn’t observe such association regarding mental component. Similar findings have been previously reported in several studies [35, 36].

It is well-documented that CAD, DM and HTN is not only associated with increased morbidity and mortality but also with substantial impairment in HRQoL [37]. Martin Fortin et al. in a systematic review showed a negative effect of multimorbidity on QOL in primary care setting [14]. In recent times, patient health status is considered important factor both as a risk factor and a health outcome. The AHA’s strategic goals is improvement in cardiovascular health By 2020 [38]. Therefore, comorbidities management programs along with cardiac symptoms management should be addressed improve health status and HR-QOL for CAD patients. Notable problem in managing patients with MM is a single disease view of clinical guidelines and protocols. Moreover, polypharmacy, multiple doctor appointment and engagement in several lifestyle modification were other issues can affect the patients’ health status and HR-QOL.

Our study involved some limitations that should be considered. We had small number of subjects in diabetes subgroup, which decrease the statistical power. Furthermore, we didn’t considered the comorbidity duration among study population, that could influence on HRQoL state. Additionally, the glycemic control, blood pressure level and depressive mood could have affect on HRQoL, which didn’t assess in the present study. Although the location of data gathering was the referral cardiovascular hospital of our province, but majority of patients were related to rural area and lower socioeconomic region. Thus, we can not generalize our findings to total population.

## Conclusion

This study indicated that more than 50 % of CAD patients had at least one other morbidity like hypertension or diabetes. Patients with multimorbidity were younger, more often female and less educated. CAD patients with one or more other morbidities reported significantly lower PCS physical of HRQoL compared to those without any morbidities. But, regard to mental component, CAD patients with HTN had the worse score than those without any morbidity. Patients Presence of multimorbidity in less educated patients cause more clinically effect on PCS compared to MCS.

## Abbreviations

ANOVA: analysis of variance
CABG: Coronary artery bypass surgery
CAD: Coronary Artery Disease
DM: Diabetes
HRQoL: Health-Related Quality of Life
HTN: Hypertension
MCS: Mental component summary
MM: Multimorbidity
PCS: Physical component summary
SF-36: 36-item short form
